# Determinants of Under-Immunization Among Children Between 0 and 59 Months in Buea Municipality, South Western Cameroon: Implications for National Immunization Campaign

**DOI:** 10.3390/healthcare13030239

**Published:** 2025-01-24

**Authors:** Jerome Nyhalah Dinga, Fred Ngwa Ngunjoh, Nicholas Tendongfor, Glory Enjong Mbah, Haowen Qin, Irshad Ahmed, Synthia Eni Muki, Stanley Dobgima Gamua, Rameshbabu Manyam, Vincent P. K. Titanji

**Affiliations:** 1 Michael Gahnyam Gbeugvat Foundation, Buea P.O. Box 63, Cameroon; 2 Biotechnology Unit, University of Buea, Buea P.O. Box 63, Cameroon; 3 Rollins School of Public Health, Emory University, Atlanta, GA 30322, USA; 4 African Vaccinology Network, Buea P.O. Box 63, Cameroon; 5 Faculty of Health Sciences, University of Buea, Buea P.O. Box 63, Cameroon; 6 Department of Biology, University of Bamenda, Bamenda P.O. Box 39, Cameroon; 7 Health Department, Balochistan AIDS Control Program, Quetta 87300, Pakistan

**Keywords:** under-immunization, urban health, Buea, vaccination determinants, routine immunization

## Abstract

**Introduction:** Under-immunization remains a major global public health concern. The World Health Organization (WHO) reports that approximately 20 million children globally are not fully vaccinated, with more than half of these children residing in Africa. African countries including Cameroon face unique challenges in achieving high vaccination coverage. This study aimed to determine the prevalence and determinants of under-immunization among children aged 0–59 months in Buea, Cameroon to contribute to effective national immunization policy. **Methods:** This cross-sectional study used the World Health Organization Behavioural and Social Determinants of vaccination tool to collect data from 438 caregivers of children aged 0–59 months in the city of Buea. Data were collected on socio-demographics, immunization factors, and health system variables for the study cohort. **Results:** It was found that 25.11% of children in urban Buea were under-immunized. Children in Buea Town were three times more likely to be under-immunized than those in Molyko (AOR = 3.0, 95% CI: 1.3–7.3, *p* = 0.013). Children of separated caregivers were 0.2 times less likely to be under-immunized than those of widowed caregivers (AOR = 0.2, 95% CI: 0.1–0.9, *p* = 0.036). Children whose caregivers did not receive unsolicited advice were 2.1 times more likely to be under-immunized (AOR = 2.1, 95% CI: 1.2–3.4, *p* = 0.006). Children living less than 1 mile from health facilities were 2.9 times more likely to be under-immunized than those living more than 10 miles away (AOR = 2.9, 95% CI: 1.1–7.5, *p* = 0.030). Children of caregivers employed in the private sector were 4.3 times more likely to be under-immunized compared to those of unemployed caregivers (AOR = 4.3, 95% CI: 1.1–16.2, *p* = 0.031). Children in non-owned/non-rented houses were 0.3 times less likely to be under-immunized compared to those in rented houses (AOR = 0.3, 95% CI: 0.1–0.9, *p* = 0.030). Children whose caregivers did not discuss vaccination concerns with healthcare workers were 0.6 times less likely to have under-immunized children (COR = 0.6, 95% CI: 0.3–0.9, *p* = 0.020). **Conclusions:** It was concluded that under-immunization is a significant public health problem in the city of Buea. Interventions that target the quality of services, community engagement, and the unique challenges faced by different caregiver groups, are needed.

## 1. Introduction

Immunization remains a cornerstone of public health strategies aimed at reducing morbidity and mortality caused by vaccine-preventable diseases, particularly in children. Vaccines have proven to be highly effective in preventing serious illnesses such as measles, polio, and diphtheria, which historically led to high rates of illness and death among young populations. By providing immunity, immunization not only protects individuals from these diseases but also helps to protect communities by reducing the overall transmission through herd immunity. As a result, immunization plays a critical role in ensuring the well-being of children and promoting long-term public health [1].

However, under-immunization continues to be a significant concern for public health authorities around the world. Many children, particularly in low-income or rural areas, do not receive the full range of vaccines required for optimal protection. Factors such as lack of access to healthcare, socio-economic barriers, misinformation about vaccines, and logistical challenges contribute to this issue. As long as gaps in immunization coverage persist, the risk of preventable diseases will remain, leading to unnecessary illness and deaths, as well as potential outbreaks that can strain healthcare systems. Addressing under-immunization is crucial to sustaining progress in the fight against vaccine-preventable diseases and safeguarding public health globally [1,2].

Africa faces a range of unique and complex challenges in achieving high vaccination coverage, making it difficult to ensure that all children are protected against vaccine-preventable diseases. Poverty remains one of the most significant barriers, as many families cannot afford healthcare services, and vaccination campaigns may not reach remote or underserved areas. Additionally, conflict and instability in several African countries disrupt healthcare infrastructure, making it difficult to maintain regular immunization programs and deliver vaccines to populations in need. Weak healthcare systems, characterized by shortages of trained healthcare workers and limited access to healthcare facilities, further hinder efforts to expand vaccination coverage. Cultural beliefs and misinformation about vaccines also contribute to resistance, as some urban communities may be wary of vaccination or prefer traditional healing practices over modern medical interventions [3,4,5].

The city of Buea in Cameroon, like other urban areas, faces unique immunization challenges compared to rural areas. These include population density and diversity, healthcare access and inequality, overcrowding and sanitation, competition for healthcare resources, cultural beliefs, and information gaps [6]. This highlights the urgency of addressing under-immunization in the urban cities [7,8]. Even though urban areas have been shown to have higher vaccination coverage compared to rural ones, mainly due to better access to healthcare facilities and more staff and greater access to vaccines, there are still specific barriers that can reduce immunization coverage compared to rural areas, including population diversity and urban poverty [9].

The Expanded Program on Immunization (EPI) in Cameroon was established in 1976 to enhance vaccination coverage for children under five years of age. Over the years, the EPI has contributed significantly to increasing vaccination rates, achieving over 80% coverage in certain periods. However, despite these advancements, the program has faced challenges in maintaining and improving coverage levels. For instance, from 2013 to 2019, the coverage of the diphtheria–tetanus–pertussis (DTP-3) vaccine dropped from 89% to 67%, leaving many children without essential vaccinations [10,11,12]. The EPI provides free vaccines against at least 14 preventable diseases, and it has been integrated into the national health system to ensure broader access. Despite the progress made, access to vaccination services remains limited in some regions, and ongoing efforts are necessary to address gaps in immunization coverage and to reach zero-dose children [11,12]. Zero-dose children are children who lack access to or are never reached by routine immunization services.

The WHO Behavioral and Social Determinants (WHO BeSD) of vaccination tool is a standardized framework to identify and assess the social and behavioral factors, beyond a person’s health, associated with healthcare interventions, hence, providing evidence-based planning to address the underlying social determinants impacting their health. This study used the WHO BeSD to determine the prevalence and determinants of under-immunization among children aged 0–59 months in the urban settings of Buea in Cameroon to inform policy.

## 2. Methods

### 2.1. Study Design and Setting

This was a cross-sectional study to capture data on the prevalence and determinants of under-immunization. The city of Buea is made up of 140,533 people as of 2024 located in five major quarters: Bokwango, Buea Road, Buea Town, Great Soppo, and Molyko [13]. The study focused on the quarters of Buea Road, Molyko, Bokwango, and Buea Town, comprising 22 communities. The urban landscape encompasses a mix of residential, commercial, and healthcare infrastructures, providing a dynamic backdrop for understanding childhood immunization in an urban context.

### 2.2. Data Collection and Sampling

The study focused on caregivers of children aged 0–59 months in urban Buea, a critical age for immunization. Using Cochran’s formula, the sample size was calculated to be 385, but data were collected from 438 participants.

Multistage sampling was performed to ensure a representative sample. This was achieved by breaking down the four quarters into smaller, more manageable clusters, communities, and study cohorts. Purposive sampling selected Urban Health Areas (quarters) in Buea, and probability proportionate to size to select participants per health area. Simple random sampling was used to choose 22 communities, cluster sampling was used to create clusters within communities, and simple random sampling was used to select households from each cluster.

A primary caregiver is fully responsible for the welfare of the child while a secondary caregiver will assist and fill in whenever the primary caregiver is unavailable. Primary caregivers of eligible children (0–59 months) who gave their consent were included in the study. Those who were extremely sick were excluded from the study. Structured questionnaires were used to collect data on socio-demographics, immunization factors, and health system variables.

### 2.3. Sample Size Calculations

The sample size for this study was calculated using the Crochan formula [14,15]:

n = (Z^2^ × p × (1 − p))/E^2^,

where;

- n: is the required sample size,
- Z^2^: is the z-score corresponding to the desired confidence level (e.g., 1.96 for 95% confidence),
- p: is the estimated proportion of the population with the characteristic of interest (if unsure, use 0.5 for maximum variability),
- E^2^: is the desired margin of error (level of precision),

n = 385 participants needed.

### 2.4. Data Management and Analysis

The structured questionnaire was based on the World Health Organization Behavioural and Social Determinants (WHO BeSD) of vaccination tool. It is preferred because it provides a well-rounded, evidence-based, and context-sensitive framework for understanding and addressing the behavioral and social factors influencing vaccination decisions. The questionnaire was pretested and adjusted for clarity and relevance by having 25 individuals from the study site fill out the questionnaire followed by a cognitive interview.

Data were collected electronically via Google Forms and entered by trained personnel. Analysis was performed using SPSS version 26, with descriptive statistics summarizing the study population and vaccination coverage. Prevalence of under-immunization and zero dose were calculated, and chi-square tests and logistic regression identified significant predictors, with a *p*-value < 0.05 considered significant.

### 2.5. Ethical Considerations

Ethical clearance was obtained from the IRB at the Faculty of Health Sciences, University of Buea, ref no: ref: 2024/2359-01/UB/SG/IRB/FHS of 14 February 2024. The objectives of the study were explained to the caregivers of eligible children and the signed consent form was obtained before the questionnaire was administered.

## 3. Results

### 3.1. Socio-Demographic Characteristics of Study Participants

Most of the participants (41.3%) were from Buea Road. Caregivers had a mean age of 31 years, mostly women (87.7%), aged 22–30 years (56.2%) (Table 1). Children’s ages were mainly 25–36 months (29.9%) and 0–12 months (28.8%). Most caregivers were married (49.5%), university-educated (53.7%), self-employed (56.2%), and had one child (56.8%). Monthly income was below 50,000 FCFA by 42.9%. The study population was predominantly Christian (94.3%) and leaseholders (74.2%). Table 1 shows the sociodemographic characteristics of participants.

### 3.2. Attitudes and Vaccination Practices of Study Participants

This present study found that 74.4% of children received all recommended vaccines, with 80.6% of caregivers providing vaccination cards. Most children (78.3%) had not contracted vaccine-preventable diseases (Table 2). However, only 59.4% of caregivers discussed vaccination concerns with healthcare workers. Family influence was significant for 53.2% of caregivers, while 28.3% faced societal stigma for not vaccinating. Additionally, 66.7% had never received unsolicited vaccination advice, and 78.8% did not hold positive cultural beliefs about vaccines. Table 2 shows the distribution of attitudes and vaccination practices among study participants in the Buea municipality.

### 3.3. Prevalence of Under-Immunization

**Figure 1:**
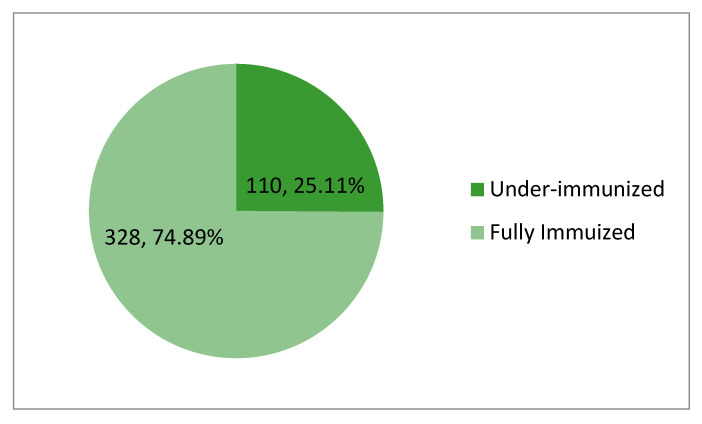
Prevalence of under-immunization.

The proportion of participants who have received some, but not all vaccine doses was calculated by (number of under-immunized participants/total number of participants) × 100%; (110/438) × 100% = 25.11% (Figure 1).

Therefore, the prevalence of under-immunization in this study was 25.11%.

### 3.4. Behavioral and Health System Factors and Their Distribution Among Caregivers of Children

The present study found that 292 (66.7%) caregivers rarely discussed vaccines with healthcare providers, though 77 (17.6%) had positive cultural practices regarding vaccination. About 173 (39.5%) lived within 1 mile of a health facility, but 229 (52.3%) experienced vaccine stockouts (Table 3). Waiting times were moderate for 219 (50%), and 291 (66.4%) received vaccination reminders. Most of the participants (291 (66.4%)) did not perceive changes in vaccine quality, suggesting it may not significantly impact vaccination decisions. Table 3 shows the behavioral and health system characteristics of caregivers surveyed in the present study.

### 3.5. Association Between Social Factors and Under-Immunization

Bivariate analysis showed that caregivers who did not receive unsolicited advice from peers were 1.7 times more likely to have under-immunized children (cOR = 1.7, 95% CI: 1.1–2.6, *p* = 0.025) than those who did. Also, the children of caregivers without positive cultural beliefs about vaccines were 1.9 times more likely to be under-immunized (cOR = 1.9, 95% CI: 1.2–3.0, *p* = 0.014) than their counterparts (Table 4).

### 3.6. Association Between Accessibility, Acceptance, and Utilization of Vaccination Services and Under-Immunization

Table 5 shows caregivers without supportive cultural practices were 1.9 times more likely to have under-immunized children (cOR = 1.9, 95% CI: 1.1–3.2, *p* = 0.018) than those with supportive cultural practices. Those who lived further away were more likely to have under-immunized children than those living nearby. Caregivers living less than 1 mile from health facilities were 3.3 times less likely to have under-immunized children (cOR = 3.3, 95% CI: 1.4–7.6, *p* = 0.005) than those living more than 10 miles away. Those who did not perceive improvements in vaccine quality were 1.7 times more likely to have under-immunized children (cOR = 1.7, 95% CI: 1.1–2.7, *p* = 0.016) than caregivers who perceived improvement in the quality of vaccines and services (Table 5). Table 5 shows the factors that are associated with immunization coverage.

### 3.7. Association Between Socio-Demographic Factors and Under-Immunization

Multivariable analysis found that participants who lived in Buea Town were 2.6 times more likely to be under-immunized than those in Molyko (adjusted odds ratio (aOR) = 2.6, 95% CI: 1.2–5.7, *p* = 0.016). Those with one child were 1.7 times more likely to have under-immunized children than those with two or more children (aOR = 1.7, 95% CI: 1.0–2.7, *p* = 0.041). Children of caregivers who were educated up to the primary level, were 2.1 times more likely to be under-immunized than those of caregivers who had a university education (Table 6). Caregivers not living in rented or owned houses were 0.3 times less likely to have under-immunized children than those in rented houses (aOR = 0.4, 95% CI: 0.1–0.7, *p* = 0.005) (Table 6).

### 3.8. Socio-Cultural and Healthcare Accessibility Factors That Are Associated with Under-Immunization

Table 7 shows the multivariable analysis which found that children in Buea Town were 3 times more likely to be under-immunized than those in Molyko (aOR = 3.0, 95% CI: 1.3–7.3, *p* = 0.013). While children of separated caregivers were 0.2 times less likely to be under-immunized than those of widowed caregivers (aOR = 0.2, 95% CI: 0.1–0.9, *p* = 0.036). It was observed that children of private sector-employed caregivers were 4.3 times more likely to be under-immunized than those of unemployed caregivers (aOR = 4.3, 95% CI: 1.1–16.2, *p* = 0.031). Children in non-owned/non-rented houses were 0.3 times less likely to be under-immunized than those in rented houses (aOR = 0.3, 95% CI: 0.1–0.9, *p* = 0.030). Children whose caregivers did not receive unsolicited advice were 2.1 times more likely to be under-immunized (aOR = 2.1, 95% CI: 1.2–3.4, *p* = 0.006). It was also observed that children living less than 1 mile from health facilities were 2.9 times more likely to be under-immunized than those living more than 10 miles away (aOR = 2.9, 95% CI: 1.1–7.5, *p* = 0.030). Children whose caregivers did not perceive improvements in vaccine services were 1.9 times more likely to be under-immunized (aOR = 1.9, 95% CI: 1.1–3.2, *p* = 0.021) than those who perceived improvements in vaccine services (Table 7).

## 4. Discussion

The prevalence of under-immunization among children aged 0–59 months in the present study was found to be 25.11%. This figure contrasts with findings from a study conducted in the Foumban Health District of Cameroon, which reported a significantly higher vaccination coverage of 80% for the DPT-HiB+HB (diphtheria, pertussis, tetanus, Haemophilus influenzae type b, and hepatitis B) vaccine among children aged 0–59 months in Foumban [16] suggests that under-immunization may vary geographically, with certain regions exhibiting better immunization coverage than others. Furthermore, a separate study focused on hepatitis B vaccination uptake among healthcare workers in Cameroon revealed a vaccination rate of 27.4% [17]. This is lower than the 25.11% under-immunization rate identified among children in the current study. This comparison suggests that the prevalence of under-immunization is somewhat lower among children aged 0–59 months than it is among healthcare workers in Cameroon, indicating that healthcare workers may face unique barriers to vaccination, such as occupational risk factors, vaccine availability, or awareness issues, that differ from those affecting children. These findings highlight the importance of considering both demographic and geographical factors when assessing vaccination coverage and under-immunization rates, as well as the potential disparities between different population groups, including children and healthcare workers. Addressing these disparities will be crucial in achieving universal immunization coverage and reducing preventable diseases in both child and adult populations.

Children in Buea Town were found to be three times more likely to be under-immunized compared to those in Molyko (AOR = 3.0, 95% CI: 1.3–7.3, *p* = 0.013). This result is consistent with findings from various studies, which have highlighted that immunization rates can vary significantly within urban areas, often due to differences in healthcare access, socio-economic conditions, and levels of community engagement. For example, a study conducted in Addis Ababa, Ethiopia, demonstrated that urban areas with poorer infrastructure and lower socio-economic status had significantly lower vaccination coverage rates compared to wealthier neighborhoods [18]. Similarly, studies in India have shown that while urban areas may generally have greater access to healthcare services, factors like slum dwelling, low income, and educational barriers often result in lower immunization rates in specific urban pockets [19]. These findings suggest that specific barriers in Buea Town, such as limited healthcare access, socio-economic disparities, and lower levels of community engagement, may contribute to the higher likelihood of under-immunization observed in this study. In urban centers, healthcare infrastructure can be uneven, with certain areas, especially informal settlements or economically disadvantaged neighborhoods, facing difficulties in accessing quality healthcare services [20]. Socio-economic conditions also play a significant role, as families with lower incomes or lower levels of education may face greater challenges in accessing vaccines or may not be fully aware of the importance of immunization [21]. Furthermore, gaps in community engagement and health promotion efforts can also lead to vaccine hesitancy or a lack of information about vaccination services, particularly in urban areas with diverse populations and high rates of migration [22]. Addressing these barriers requires targeted interventions that focus on improving healthcare access, strengthening community-based health initiatives, and addressing socio-economic inequalities. Moreover, increasing outreach efforts to engage underserved populations in Buea Town, ensuring equitable distribution of healthcare resources, and enhancing public health education campaigns could help reduce the under-immunization rate observed in this study. By addressing these underlying factors, public health authorities can work towards achieving higher immunization coverage and improving health outcomes for children in both Buea Town and other urban areas facing similar challenges.

The finding that children in Buea Town are three times more likely to be under-immunized compared to those in Molyko is in line with studies showing urban areas can exhibit varied immunization rates due to multiple factors such as healthcare infrastructure, socio-economic conditions, and cultural practices. For instance, a study in sub-Saharan Africa highlighted that urban areas, while often having more healthcare facilities, may experience disparities in immunization uptake due to overcrowding, inefficiency in service delivery, or issues related to migrant populations who may be less informed about the available services, and areas that might have lower access to healthcare facilities but tighter-knit communities that foster more consistent vaccination behaviors [23]. Addressing barriers in Buea Town, such as improving service delivery, ensuring equitable access to healthcare, and strengthening community engagement, could be key to addressing these disparities.

The observation that children of caregivers who were separated from their spouse are less likely to be under-immunized compared to those with widowed caregivers contradicts the expected trend that single-parent households, particularly those headed by widows, are more likely to face barriers to healthcare. This could reflect differences in the support systems available to separated versus widowed caregivers. Research has shown that widowed caregivers may face heightened socio-economic vulnerabilities, which could limit their ability to prioritize and access healthcare for their children [24]. In contrast, separated caregivers might benefit from broader family or social support networks that help ensure immunization adherence, even if they are single. However, further investigation into the dynamics of single-parent households in this context is warranted to fully understand the observed trend [25].

Children of caregivers employed in the private sector are more likely to be under-immunized than the children of unemployed caregivers, which supports the general expectations that employment increases access to healthcare. Several studies have found that employed caregivers, especially those in the public sector, often have better healthcare access due to benefits like paid leave, healthcare insurance, and better economic stability [26]. However, the demanding nature of private sector jobs could create time constraints for caregivers, limiting their ability to take their children for vaccinations. This underscores the importance of considering both the economic benefits of employment and the time-related challenges posed by different work sectors when addressing immunization barriers.

The caregivers not living in their own house or rented property with children were less likely to be immunized compared to those leaving in rented houses. This presents a unique finding suggesting that housing stability may play a role in ensuring regular healthcare visits and adherence to vaccination schedules. This contrasts with the idea that renters or homeowners are more likely to have stable living conditions conducive to healthcare access. One possible explanation could be that families in non-rented housing such as those staying with relatives or in temporary housing, might benefit from more flexible or informal support networks that could aid in regular healthcare visits. Alternatively, this finding may reflect socio-economic factors tied to housing types that influence health-seeking behavior [27].

It was more common to see under-immunized children with caregivers who did not receive unsolicited advice compared to their counterpart. This aligns with research emphasizing the significant role of social networks in health behaviors. Studies have shown that peer influence and community engagement can strongly affect health decisions, including immunization adherence [28]. Caregivers who are not exposed to informal key information about immunization schedules, the importance of vaccines, or how to access healthcare services are more likely to have under-immunized children. This highlights the value of public health initiatives that leverage community-based outreach and peer support to improve vaccination rates.

The children living less than 1 mile from health facilities were more likely to be under-immunized. This contradicts the general assumption that proximity to healthcare services improves vaccination rates. In theory, closer proximity should make it easier for caregivers to access services. However, this result suggests that proximity alone is insufficient to explain immunization uptake. Other factors, such as the quality of healthcare services, healthcare worker attitudes, financial barriers, and perceived healthcare system trust, may play a more significant role. Studies have shown that even when healthcare services are geographically accessible, they may not be utilized if there are issues with service delivery, such as long wait times or negative experiences with healthcare workers [29].

Caregivers who did not perceive improvements in vaccine services were more likely to have under-immunized children compared to those who did. This is consistent with the literature highlighting the importance of trust in healthcare services for improving vaccination compliance [30]. Caregivers who believe that healthcare services have not improved may be less motivated to seek immunizations for their children, as they may perceive these services as inadequate or unreliable. Perceived quality of healthcare plays a critical role in health-seeking behaviors and improving this perception could lead to better immunization outcomes [30].

## 5. Conclusions

These findings offer crucial insights into the multifaceted barriers to immunization in the municipality of Buea, highlighting the need to address socio-economic disparities, healthcare access challenges, and the role of social networks in shaping health behaviors. Tailored interventions must not only focus on proximity to healthcare facilities but also improve service quality, engage communities, and consider the distinct challenges faced by different caregiver groups.

However, the study’s limitations, including recall bias and its cross-sectional nature, suggest that further research is needed to deepen our understanding of vaccine uptake trends. Conducting qualitative studies will be essential for uncovering the underlying reasons for under-vaccination, particularly in missed or marginalized communities. Given the specific context of this study in Cameroon, broader research is needed to determine whether these findings are applicable nationwide, especially in light of the impacts of civil conflict and the COVID-19 pandemic. These insights can guide the development of more effective, targeted vaccination strategies to enhance public health outcomes.

## Figures and Tables

**Table 1:** Socio-Demographic Characteristics of Study Population.

Variable	Category	Frequency (Percentage)
Quarter	Bokwango	61 (13.9%)
	Buea Road	181 (41.3%)
	Buea Town	66 (15.1%)
	Molyko	130 (29.7%)
	**Total**	**438 (100%)**
**Community**		
	Bokwai Layout	19 (4.3%)
	Bokwango	14 (3.2%)
	Bonalyonga	12 (2.7%)
	Buea Station	29 (6.6%)
	Campsic	11 (2.5%)
	Check Point	32 (7.3%)
	GRA	12 (2.7%)
	Great Soppo	35 (8.0%)
	Likoko	12 (2.7%)
	Long Street	15 (3.4%)
	Lower Bonduma	51 (11.6%)
	Malingo	30 (6.8%)
	Mukunda	11 (2.5%)
	Naanga	12 (2.7%)
	Ndongo	21 (4.8%)
	Sandpit	10 (2.3%)
	Stranger East	9 (2.1%)
	Stranger west	1 (0.2%)
	Stranger West	18 (4.1%)
	UB 1 and 2	28 (6.4%)
	Upper Bonduma	41 (9.4%)
	Wondongo	15 (3.4%)
	**Total**	**438 (100%)**
**Ages of Caregivers (years)**		
	22–30	246 (56.2%)
	31–40	137 (31.3%)
	41–50	47 (10.7%)
	51–60	8 (1.8%)
	**Total**	**438 (100%)**
**Ages of Children (months)**		
	0–12	126 (28.8%)
	13–24	90 (20.5%)
	25–36	131 (29.9%)
	37–48	48 (11.0%)
	51–59	43 (9.8%)
	**Total**	**438 (100%)**
**Sex of caregiver**		
	Female	384 (87.7%)
	Male	54 (12.3%)
	**Total**	**438 (100%)**
**Marital Status**		
	Divorced	11 (2.5%)
	Married	217 (49.5%)
	Separated	21 (4.8%)
	Single	172 (39.3%)
	Widowed	17 (3.9%)
	**Total**	**438 (100%)**
**Level of Education**		
	No Formal Education	12 (2.7%)
	Primary	29 (6.6%)
	Secondary	162 (37.0%)
	University	235 (53.7%)
	**Total**	**438 (100%)**
**Occupation**		
	Employed (Government)	54 (12.3%)
	Employed (Private Sector)	25 (5.7%)
	Self-employed (Business)	246 (56.2%)
	Unemployed	113 (25.8)
	**Total**	**438 (100%)**
**Number of Children**		
	More than Two	74 (16.9%)
	One	249 (56.8)
	Two	115 (26.3%)
	**Total**	**438 (100%)**
**Monthly Income (FCFA/XAF)**		
	Less than 50 K	188 (42.9)
	50 K–99 K	145 (33.1%)
	100 K–150 K	69 (15.8%)
	Above 150 K	36 (8.2%)
	**Total**	**438 (100%)**
**Religion**		
	Christian	413 (94.3%)
	Muslim	11 (2.5%)
	Others	14 (3.2%)
	**Total**	**438 (100%)**
**Caregiver Status**		
	Primary caregiver	250 (57.1%)
	Secondary caregiver	54 (12.3%)
	Shared responsibility	134 (30.6)
	**Total**	**438 (100%)**
**Housing Situation**		
	Rented	325 (74.2%)
	Owned	86 (19.6%)
	Other	27 (6.2%)
	**Total**	**438 (100%)**

**Table 2:** Distribution of attitudes and vaccination practices among study participants.

Variable	Category	Frequency (Percentage)
**Child Vaccination**		
	Have not received a single dose	2 (0.5%)
	No, received some.	110 (25.1%)
	Yes, received all.	326 (74.4%)
	**Total**	**438 (100%)**
**Presence of Vaccination Card**		
	No	85 (19.4%)
	Yes	353 (80.6%)
	**Total**	**438 (100%)**
**Child Experienced Vaccine-preventable Disease VPDs**		
	No	343 (78.3%)
	Yes	95 (21.7%)
	**Total**	**438 (100%)**
**Discussed Vaccination Concerns with Healthcare workers**		
	No	178 (40.6%)
	Yes	260 (59.4%)
	**Total**	**438 (100%)**
**Influence of Family**		
	No	209 (47.7%)
	Yes	229 (47.7%)
	**Total**	**438 (100%)**
**Societal Stigma**		
	No	314 (71.7%)
	Yes	124 (28.3%)
	**Total**	**438 (100%)**
**Received Advice**		
	No	292 (66.7%)
	Yes	146 (33.3%)
	**Total**	**438 (100%)**
**Good Cultural Beliefs**		
	No	345 (78.8%)
	Yes	93 (21.2%)
	**Total**	**438 (100%)**

**Table 3:** Distribution of Behavioral and Use of Health System Characteristics Amongst the Caregivers.

Variable	Category	Frequency (Percentage)
**Frequency of Vaccine Discussions**		
	Frequently	146 (33.3%)
	Rarely	292 (66.7%)
	**Total**	**438 (100%)**
**Cultural Practices**		
	No	361 (82.4%)
	Yes	77 (17.6%)
	**Total**	**438 (100%)**
**Proximity to Health Facility**		
	1–5 miles	159 (36.3%)
	5–10 miles	78 (17.8%)
	Less than 1 mile	173 (39.5%)
	More than 10 miles	28 (6.4%)
	**Total**	**438 (100%)**
**Experienced Vaccine Stock outs**		
	No	229 (52.3%)
	Yes	209 (74.7%)
	**Total**	**438 (100%)**
**Waiting Time**		
	Long	116 (26.5%)
	Moderate	219 (50%)
	Short	103 (23.5%)
	**Total**	**438 (100%)**
**Reminders or Notifications**		
	No	147 (33.6%)
	Yes	291 (66.4%)
	**Total**	**438 (100%)**
**Perceived Change in Quality of Vaccine and Services**		
	No	291 (66.4%)
	Yes	147 (33.6%)
	**Total**	**438 (100%)**

**Table 4:** Association Between Social Factors and Under-immunization in the Study Participants of the Buea Municipality.

		No	Yes	Total	cOR	95% CI		
						Lower	Upper	*p*-Value
Child Experienced Vaccine Preventable Disease (VPDs)	No	262	81	343	1.567	0.954	2.573	0.076
	Yes	64	31	95	1			
Discussed Vaccination Concerns With Healthcare workers	No	122	56	178	0.598	0.388	0.922	0.020
	Yes	204	56	260	1			
Influence of Family	No	151	58	209	0.803	0.523	1.235	0.318
	Yes	296	90	386	1			
Societal Stigma	No	240	74	314	1.433	0.903	2.275	0.127
	Yes	86	38	124	1			
Received Unsolicited Advice	No	227	65	292	1.658	1.064	2.583	0.025
	Yes	99	47	146	1			
Cultural Beliefs	No	266	79	345	1.852	1.131	3.033	0.014
	Yes	60	33	93	1			

**Table 5:** Accessibility, Acceptance, and Utilization of Vaccination Services Factors Associated with Under-immunization.

		No	Yes	Total	cOR	95% CI		
						Lower	Upper	*p*-Value
Frequency of Vaccine Discussions	Frequently	116	30	146	1.510	0.938	2.430	0.090
	Rarely	210	82	292	1			
Cultural Practices	No	277	84	361	1.884	1.115	3.184	**0.018**
	Yes	49	28	77	1			
Proximity to Health Facility	1–5 miles	117	42	159	2.414	1.061	5.493	**0.036**
	5–10 miles	57	21	78	2.352	0.961	5.760	0.061
	Less than 1 mile	137	36	173	3.298	1.440	7.552	**0.005**
	More than 10 miles	15	13	28	1			
Experienced Vaccine Stockouts	No	167	62	229	0.847	0.550	1.304	0.450
	Yes	159	50	209	1			
Waiting Time	Long	82	34	116	0.900	0.499	1.624	0.727
	Moderate	169	50	219	1.262	0.738	2.158	0.396
	Short	75	28	103	1			
Reminders or Notifications	No	106	41	147	0.834	0.533	1.307	0.429
	Yes	220	71	291	1			
Perceived Change in Quality of Vaccine and Services	No	227	64	291	1.720	1.105	2.677	**0.016**
	Yes	99	48	147	1			

**Table 6:** Socio-Demographics Factors that Influence Under-immunization.

		No	Yes	Total	aOR	95% CI		
						Lower	Upper	*p*-Value
Health Area	Bokwango	45	16	61	1.296	0.656	2.557	0.455
	Buea Road	136	45	181	1.392	0.844	2.297	0.195
	Buea Town	56	10	66	2.580	1.197	5.560	0.016
	Molyko	89	41	130	1			
Age of Caregiver	22–30	171	75	246	0.00	0.00	0.00	0.999
	31–40	109	28	137	0.00	0.00	0.00	0.999
	41–50	38	9	47	0.00	0.00	0.00	0.999
	51–60	8	0	8	1.00			
Age of Child	0–12	95	31	126	1.479	0.695	3.150	0.310
	13–24	68	22	90	1.492	0.671	3.317	0.326
	25–36	95	36	131	1.274	0.605	2.682	0.524
	37–48	39	9	48	2.092	0.797	5.494	0.134
	51–59	29	14	43	1			
Sex of caregiver	Female	286	98	384	1.021	0.533	1.957	0.949
	Male	40	14	54	1			
Marital Status	Divorced	8	3	11	1.455	0.277	7.637	0.658
	Married	176	41	217	2.341	0.818	6.699	0.113
	Separated	7	14	21	0.273	0.071	1.048	0.059
	Single	124	48	172	1.409	0.494	4.023	0.522
	Widowed	11	6	17	1			
Level of Education	No Formal Education	7	5	12	0.480	0.147	1.569	0.225
	Primary	25	4	29	2.143	0.717	6.408	0.173
	Secondary	119	43	162	0.949	0.602	1.496	0.821
	University	175	60	235	1			
Occupation	Employed (Government)	45	9	54	2.338	1.032	5.296	**0.042**
	Employed (Private Sector)	21	4	25	2.455	0.785	7.676	0.123
	Self-employed (Business)	183	63	246	1.358	0.833	2.213	0.219
	Unemployed	77	36	113	1			
Number of Children	More than Two	54	20	74	1.281	0.672	2.442	0.452
	One	194	55	249	1.673	1.022	2.738	**0.041**
	Two	78	37	115	1			
Monthly Income (FCFA/XAF)	100 K–150 K	56	13	69	1.561	0.787	3.095	0.203
	50 K–99 K	103	42	145	0.889	0.548	1.440	0.632
	Above 150 K	29	7	36	1.501	0.619	3.643	0.369
	Less than 50 K	138	50	188	1			
Religion	Christian	307	106	413	0.483	0.106	2.192	0.345
	Muslim	7	4	11	0.292	0.042	2.023	0.212
	Others	12	2	14	1			
Caregiver Status	Secondary Caregiver	30	24	54	0.353	0.191	0.652	**0.001**
	Shared Responsibility	101	33	134	0.863	0.527	1.415	0.560
	Primary caregiver	195	55	250	1			
Housing Situation	Other	13	14	27	0.318	0.144	0.705	**0.005**
	Owned	71	15	86	1.623	0.882	2.988	0.120
	Rented	242	83	325	1			

**Table 7:** Association between Socio-Cultural and Healthcare Accessibility Factors and Under-immunization.

Variable	Category	aOR		95% CI		
				Lower	Upper	*p*-Value
	(Intercept)	0.737		0.062	8.778	0.809
**Health Area**	Bokwango	0.931		0.418	2.073	0.862
	Buea Road	1.706		0.941	3.093	0.078
	Buea Town	3.046		1.270	7.306	**0.013**
	Molyko	1				
**Marital Status**	Divorced	2.310		0.348	15.330	0.386
	Married	2.628		0.721	9.576	0.143
	Separated	0.188		0.040	0.895	**0.036**
	Single	1.899		0.515	6.998	0.335
	Widowed	1				
**Occupation**	Employed (Government)	2.348	0.846		6.519	0.101
	Employed (Private Sector)	4.295	1.139		16.196	**0.031**
	Self-employed (Business)	1.423	0.792		2.559	0.238
	Unemployed	1				
**Religion**	Christian	0.184	0.029		1.173	0.073
	Muslim	0.105	0.011		1.048	0.055
	Others	1				
**Caregiver Status**	No, another family member is the primary caregiver	0.481	0.229		1.008	0.053
	Shared care giving responsibility	0.554	0.305		1.008	0.053
**Housing Situation**	Other	0.346	0.133		0.900	**0.030**
	Owned	1.623	0.761		3.462	0.210
	Rented	1				
**Child Experienced Vaccine** **Preventable Disease (VPD)**	No	1.564	0.872		2.806	0.134
	Yes	1				
**Discussed Vaccination** **Concerns with healthcare workers**	No	0.603	0.358		1.016	0.058
	Yes	1				
**Received Unsolicited** **Advice**	No	2.055	1.232		3.429	**0.006**
	Yes	1				
**Frequency of** **Vaccine Discussion**	Frequently	1.676	0.953		2.946	0.073
	Rarely	1				
**Proximity to** **Health Facility**	1–5 miles	2.105	0.810		5.469	0.126
	5–10 miles	2.769	0.961		7.976	0.059
	Less than 1 mile	2.887	1.109		7.511	**0.030**
	More than 10 miles	1				
**Perceived change** **in Quality of Vaccine** **services**	No	1.881	1.100		3.216	**0.021**
	Yes	1				

## Data Availability

Data used for this research are available from the corresponding author upon request from the corresponding author.
